# Alkaloid Profiling and Anti-Cholinesterase Potential of Three Different Genera of Amaryllidaceae Collected in Ecuador: *Urceolina* Rchb., *Clinanthus* Herb. and *Stenomesson* Herb.

**DOI:** 10.3390/life14080924

**Published:** 2024-07-24

**Authors:** Luciana R. Tallini, Karen Acosta León, Raúl Chamorro, Edison H. Osorio, Jaume Bastida, Lou Jost, Nora H. Oleas

**Affiliations:** 1 Grup de Productes Naturals, Departament de Biologia, Sanitat i Medi Ambient, Facultat de Farmàcia i Ciències de l’Alimentació, Universitat de Barcelona, Av. Joan XXIII 27-31, 08028 Barcelona, Spain; ruscheltallini@ub.edu (L.R.T.); jaumebastida@ub.edu (J.B.); 2 Grupo de Investigación de Productos Naturales y Farmacia, Facultad de Ciencias, Escuela Superior Politécnica del Chimborazo, Panamericana Sur km 1 1/2, Riobamba EC060155, Ecuador; karen.acosta.leon@gmail.com (K.A.L.);; 3 Facultad de Ciencias Naturales y Matemáticas, Universidad de Ibagué, Carrera 22 Calle 67, Ibagué 730002, Colombia; 4 Fundación EcoMinga, Vía a Runtún s/n, Baños EC180250, Ecuador; 5 Centro de Investigación de la Biodiversidad y Cambio Climático (BioCamb) y Facultad de Ciencias de la Salud y Bienestar Humano, Universidad Tecnológica Indoamérica, Machala y Sabanilla, Quito EC170301, Ecuador

**Keywords:** alkaloids, Alzheimer’s disease, Amaryllidoideae, Amazon lily, computational experiments

## Abstract

Ecuador is an important center of biodiversity for the plant subfamily Amaryllidoideae, known for its important bioactive molecules. This study aimed to assess the chemical and biological potential of four different Amaryllidoideae species collected in Ecuador: *Urceolina formosa*, *Urceolina ruthiana*, *Clinanthus incarnatus*, and *Stenomesson aurantiacum*. Twenty-six alkaloids were identified in the bulb extracts of these species using GC-MS. The extract of *S. aurantiacum* exhibited the greatest structural diversity and contained the highest amounts of alkaloids, particularly lycorine and galanthamine. Only for this species, identification of all the alkaloids belonging to this chemical profile was not possible. Six of them remain unidentified. The potential of these three Amaryllidoideae genera against Alzheimer’s disease was then evaluated by measuring their AChE and BuChE inhibitory activity, revealing that *C. incarnatus* and *U. formosa* (from Sucumbíos province) showed the best results with IC_50_ values of 1.73 ± 0.25 and 30.56 ± 1.56 µg·mL^−1^, respectively. Molecular dynamic assays were conducted to characterize the possible interactions that occurs among 2-hydroxyanhydrolycorine and the AChE enzyme, concluded that it is stabilized in the pocket in a similar way to galanthamine. This study expands our understanding of the biodiversity of Amaryllidoideae species from Ecuador, highlighting their potential as source of chemical compounds with pharmaceutical applications.

## 1. Introduction

The Amaryllidaceae family, specifically the subfamily Amaryllidoideae, is an important source of a specific group of alkaloids, known as Amaryllidaceae alkaloids, which exhibit a broad spectrum of biological activities [1]. This subfamily comprises ca. 900 species and 75 genera, producing about 700 Amaryllidaceae alkaloids. These alkaloids are mainly classified into nine skeleton types: norbelladine-, lycorine-, homolycorine-, crinine-, haemanthamine-, narciclasine-, tazettine-, montanine-, and galanthamine-type structures [2,3].

While the subfamily Amaryllidoideae can be found worldwide, it exhibits the greatest diversity in South America, South Africa, and the Mediterranean region [2]. Cultures around the world have used Amaryllidaceae plants for centuries in traditional medicine, recognizing their biological potential often linked to the production of specific alkaloids [4].

Ecuador is the major center of diversity in the tribe Eucharideae (Amaryllidaceae) [5], which includes the genus *Urceolina* Planch. This genus, known as the Amazon lily, is mainly distributed in the western Amazon basin and the adjoining lower slopes of the eastern Andean cordillera [6]. The genus *Stenomesson* Herb. (Amaryllidaceae) is primarily found in Peru, with only *S. aurantiacum* Herb. reported in Ecuador [7]. These plants usually occur in seasonally dry, grassy vegetation or at the margins of cloud forests above 2000 m elevation, but are also found in Peruvian inter-Andean valleys below 2000 m [8] and in the loma formations along the coast of this country. All the petiolate-leafed Stenomesseae are more closely related to Eucharideae than to the lorate-leafed Stenomesseae [5,9]. The genus *Clinanthus* Herb. (Amaryllidaceae), endemic to Peru and Ecuador [10], was separated from *Stenomesson* and is primarily known in its same locations [8,11,12].

Regarding the traditional use of these plants, *Urceolina* species have been documented as being used in compresses applied to sores and tumors by native people in Ecuador, while the Jíbaro indigenous people in Peru have employed them for treating facial blemishes and acne [13]. Additionally, archeological findings at Inca ruins in South America suggest that certain Amaryllidaceae genera, such as *Stenomesson*, were depicted on ceremonial drinking vessels, indicating their significance in popular culture [8,14]. In recent years, the Amaryllidaceae species from Ecuador have garnered significant attention due to their alkaloid profiling and potential therapeutic benefits against Alzheimer’s disease. The description and understanding of these species have progressively expanded in the literature in the last decade [15,16,17,18].

Dementia is a significant contributor to disability and dependency in the elderly population [19]. This condition gives rise to physical, psychological, social, and economic impacts, with Alzheimer’s disease being the most prevalent form [19]. The development of Alzheimer’s disease appears to involve various mechanisms, one of which is a decrease in the neurotransmitter acetylcholine in the inter-synaptic space [20]. Currently, galanthamine, donepezil, and rivastigmine are the only cholinesterase inhibitor drugs approved by FDA for the clinical treatment of mild to moderate Alzheimer’s disease symptoms. Galanthamine, an Amaryllidaceae alkaloid originally isolated from *Galanthus woronowii* in 1952, has been commercialized in its salt form since 2001 [21]. However, synthesizing this molecule remains expensive; thus, pharmaceutical companies continue to extract it from Amaryllidaceae species [22].

The aim of this study was to evaluate the chemical and biological potential of five samples of Amaryllidaceae collected in Ecuador. Alkaloid extracts from the bulbs of *Urceolina formosa* Meerow, *Urceolina ruthiana* L. Jost, Oleas and Meerow, *Clinanthus incarnatus* (Kunth) Meerow, and *Stenomesson aurantiacum* (Kunth) Herb. were analyzed using gas chromatography coupled to mass spectrometry (GC-MS). Additionally, their potential in combating Alzheimer’s disease was estimated by evaluating their inhibitory activity against cholinesterase enzymes, acetylcholinesterase (AChE), and butyrylcholinesterase (BuChE). Computational experiments were also conducted to gain further insights into the molecular interactions among alkaloid and AChE.

## 2. Materials and Methods

### 2.1. Plant Material

**Figure 1:**
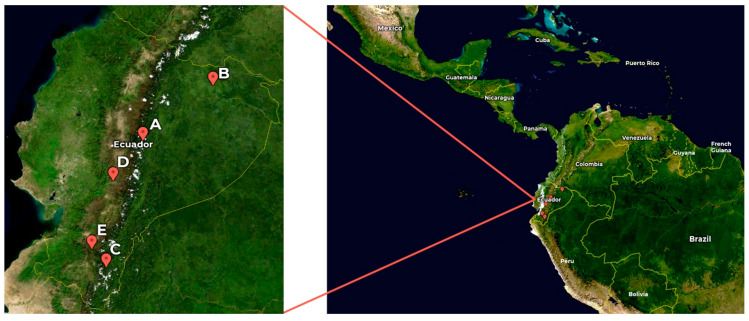
Map of Ecuador showing the sites of the samples. A: *Urceolina formosa* (from Tungurahua province); B: *Urceolina formosa* (from Sucumbíos province); C: *Urceolina ruthiana;* D: *Clinanthus incarnatus*; E: *Stenomesson aurantiacum*. Map source: NASA, Public domain, via Wikimedia Commons.

**Figure 2:**
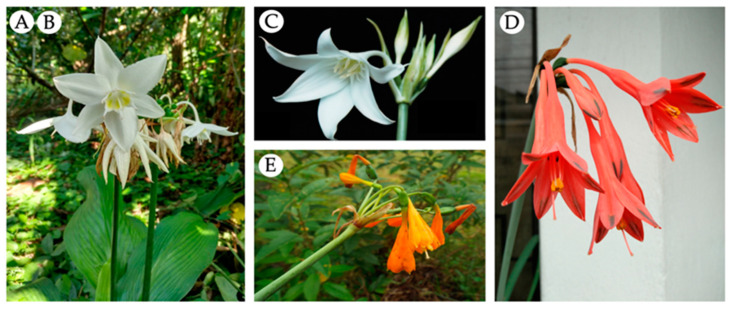
Flowering Amaryllidaceae species collected in Ecuador. A, B: *Urceolina formosa*; C: *Urceolina ruthiana*; D: *Clinanthus incarnatus*; E: *Stenomesson aurantiacum*. Pictures source: A, B: Karen Acosta C: Lou Jost, D, E: Nora H. Oleas.

Five samples of Amaryllidaceae, collected in Ecuador during 2019, were evaluated in this study (Figure 1 and Figure 2). *Urceolina formosa* (Ravenna) Ravenna (sample A) was collected in Sucumbíos province, Cantón Shushufindi, Recinto el Mirador, kilometer 5 (Acost sn, HUTI). *Urceolina formosa* (Ravenna) Ravenna (sample B) was collected in Tungurahua province, Reserva Rio Zuñag (Oleas 1049, HUTI). *Urceolina ruthiana* L. Jost, Oleas and Meerow (sample C) was obtained from Zamora-Chinchipe province, Copalinga private reserve, near Zamora (Jost 8278, QCA). *Clinanthus incarnatus* (Kunth) Meerow (sample D) was collected in Guasuntos, Chimborazo province (Oleas 43, QCA). *Stenomesson aurantiacum* (Kunth) Herb. (sample E) was collected in Loja Province, close to the border with Azuay (Meerow 1134, MO). These species were authenticated by Alan Meerow from Arizona State University, USA.

*Urceona formosa* is native to the Eastern Andes, extending towards the Amazon in Ecuador, Colombia, and Peru, at elevations between 100 to 1800 m. *Urceolina ruthiana* is endemic to Ecuador and is only known from the type locality at ca. 1100 m of elevation. *Clinathus incarnatus* is found in Ecuador and Peru between 2000 and 3000 m of elevation. *Stenomesson aurantiacum* is located along the Andes above 3000 m, from southern Colombia to northern Peru.

### 2.2. Alkaloid Extraction

The bulbs of *Urceolina*, *Clinanthus*, and *Stenomesson* species were dried at 40 °C for 7 days. Afterward, the samples were ground and the resulting powder was soaked in methanol at room temperature for 3 days, with the solvent being changed daily (3 × 100 mL). To enhance extraction efficiency, ultrasonic baths were employed for 20 min, 8 times a day. The mixture obtained was then filtered and evaporated under reduced pressure until dry. The crude bulb extracts were acidified with 30 mL of a 2% (*v*/*v*) aqueous solution of sulfuric acid to pH 2, followed by purification with diethyl ether (3 × 50 mL) into a decanting funnel to eliminate neutral components. Subsequently, the aqueous solution was alkalized with a 25% (*v*/*v*) ammonium hydroxide solution to pH 9–10 and subjected to liquid-liquid extraction with ethyl acetate (3 × 50 mL) to isolate the alkaloid extract (AE) from each sample.

### 2.3. GC-MS Analysis

Two mg of each sample were dissolved in 1 mL of methanol:chloroform (0.5:0.5, *v*/*v*) and analyzed using GC-MS (Agilent Technologies 6890N coupled with MSD5975 inert XL; Santa Clara, CA, USA) operating in electron ionization (EI) mode at 70 eV. A Sapiens-X5 MS column (30 m × 0.25 mm i.d., film thickness 0.25 µm; Teknokroma, Barcelona, Spain) was used. One µL of each sample was injected into the equipment using the splitless mode. Codeine (0.05 mg·mL^−1^) served as the internal standard. The injector and detector temperatures were set at 250 °C and 280 °C, respectively, and the flow rate of the carrier gas (He) was 1 mL·min^−1^. The temperature gradient was as follows: 12 min at 100 °C, 100–180 °C at 15 °C·min^−1^, 180–300 °C at 5 °C·min^−1^, and a 10 min hold at 300 °C.

### 2.4. Alkaloid Identification

The identification of the alkaloids present in the 5 different samples of Amaryllidaceae collected in Ecuador was carried on by comparing electro ionization (EI) fragments and Kovats Retention Indices (RI) of each compound with a comprehensive library maintained by the Natural Products Research Group within the Faculty of Pharmacy at the University of Barcelona. This extensive library, built upon over 40 years of our research group’s work, houses data for more than 300 characterized Amaryllidaceae alkaloids. The structures of these reference alkaloids were previously elucidated using various spectroscopic techniques, including Nuclear Magnetic Resonance (NMR), Ultraviolet (UV), Circular Dichroism (CD), and Mass Spectrometry (MS). The mass spectral data of these reference compounds was deconvoluted using the AMDIS 2.64 software (Automatic Mass spectral Deconvolution and Identification System, NIST) to facilitate accurate peak identification. Finally, the RI values of the compounds were determined relative to a standard n-hydrocarbon calibration mixture (C9-C36) for further characterization. Comparison with the NIST 2020 library did not yield matches for the unidentified structures.

### 2.5. Alkaloid Quantification

A calibration curve of galanthamine (10, 20, 40, 60, 80, and 100 µg·mL^−1^) was established to quantify each constituent detected in the chromatogram, using codeine (0.05 mg·mL^−1^) as the internal standard (is). Peak areas were manually determined, considering selected ions for each compound (usually the base peak of their MS, i.e., *m*/*z* at 286 for galanthamine and 299 for codeine). The ratio between the values obtained for galanthamine and codeine in each solution was plotted against the corresponding concentration of galanthamine to generate the calibration curve and its equation (y = 0.0112x − 0.0469; R^2^ = 0.9995). All data were standardized to the area of the internal standard (codeine), and the equation derived from the calibration curve of galanthamine was used to calculate the amount of each alkaloid. Results are expressed as mg equivalent GAL (galanthamine), which were then related to the alkaloid extract (AE). Since peak area depends not only on the concentration of the corresponding alkaloid but also on the intensity of the mass spectra fragmentation, the quantification is not absolute, but it is considered suitable for comparing the specific alkaloid amounts in Amaryllidaceae samples as recommended in other studies [23].

### 2.6. AChE and BuChE Inhibitory Activity

Cholinesterases inhibitory activities were determined according to Ellman et al. [24], with some modifications as described by López et al. [25]. Stock solutions containing 500U of AChE from *Electrophorus electricus* (Merck, Darmstadt, Germany) and BuChE from equine serum (Merck, Darmstadt, Germany) were prepared and stored at −20 °C. Acetylthiocholine iodide (ATCI), S-butyrylthiocholine iodide (BTCI), and 5,50-dithiobis (2-nitrobenzoic) acid (DTNB) were obtained from Merck (Darmstadt, Germany). Then, 50 µL of a work solution of 0.25 U/mL of AChE or BuChE in phosphate buffer (8 mM K_2_HPO_4_, 2.3 mM NaH_2_PO_4_, 0.15 NaCl, pH 7.5) and 50 µL of the sample dissolved in the same buffer were added to the wells. The plates were then incubated for 30 min at room temperature. Subsequently, 100 µL of the substrate solution (0.1 M Na_2_HPO_4_, 0.5 M DTNB, and 0.6 mM ATCI or 0.24 mM BTCI in Millipore water, pH 7.5) (Merck, Darmstadt, Germany) was added. After 10 min, the absorbance was read at 405 nm using a Labsystem microplate reader (Helsinki, Finland). Enzymes activities were calculated as percent compared to a control using a buffer without any inhibitor. Galanthamine served as a positive control. Initially, the activities of each sample against AChE and BuChE were assessed at concentrations of 10, 100, and 200 µg·mL^−1^. Then, the calibration curves of bulb alkaloid extracts from *Urceolina*, *Clinanthus*, and *Stenomesson* species against both enzymes were calculated and used to obtain IC_50_ values. The cholinesterase inhibitory data were analyzed with the Prism 10 software.

### 2.7. Statistical Analysis

Three independent assays were conducted to evaluate the cholinesterase activity of the alkaloid extracts from Amaryllidaceae species collected in Ecuador. The results were analyzed using ANOVA, using the Prism 10 software (GraphPad Software version 10.1.1 (270), Boston, MA, USA, www.graphpad.com). Data are presented as mean ± standard deviation (SD). Significant results are marked as follows: **** *p* < 0.0001, *** *p* < 0.001, and * *p* < 0.1. For both AChE and BuChE, one-way ANOVA with Dunnett’s multiple comparison test was used to compare the mean of each column with that of a control column (galanthamine).

### 2.8. Molecular Docking

The binding mechanisms of the alkaloids 2-hydroxyanhydrolycorine and galanthamine to the active site in *Homo sapiens* acetylcholinesterase (hAChE) protein, PDB code 4EY6 [26], were investigated using the AutoDock v.4.2 tool [27]. The use of hAChE protein has been proposed as an effective approach in the search for new cholinesterase inhibitors for human use. Water molecules, cofactors, and ions were removed from the X-ray crystallographic structure for the protein preparation procedure in the molecular docking experiment. Polar hydrogen atoms were added, and atomic charges were computed using the Gasteiger method, while the non-polar hydrogen atoms were merged. The grid maps required by AutoDock were calculated using the auxiliary program AutoGrid, choosing a 60 Å × 60 Å × 60 Å grid box around the active site. The Lamarckian Genetic Algorithm (LGA) was used to conduct the docking searches [28] using 2000 individuals as a population, resulting in 2,500,000 energy evaluations for each 100 LGA run. The best docking complex poses were analyzed based on to intermolecular interactions (ligand/enzyme), including hydrogen bonding, hydrophobic interactions, and the cation–π, π–π stacking.

### 2.9. Molecular Dynamics Simulations (MD)

MD experiments were conducted on the ligand–hAChE (PDBID:4EY6) complex in aqueous solutions containing galanthamine and 2-hydroxyanhydrolycorine as ligands, using TIP3P water model as an explicit solvent [29] (≈16.000 water molecules). Additionally, Na^+^ and Cl^−^ ions were added to neutralize the systems and maintain an ionic concentration of 0.15 mol·L^−1^. A general AMBER force field (GAFF) was used to parametrize galanthamine and 2-hydroxyanhydrolycorine molecules [30,31]. The protein structure was modeled using the CHARMM27 par_all27_prot_lipid.inp parameter [32]. The simulations followed a standard MD protocol: (I) minimization and structural relaxation of water molecules utilizing 2000 stages of minimization (downward step) and MD simulations with an NPT (310 K) assembly by 1000 ps, using harmonic constraints of 10 kcal⋅mol Å-2 on the protein and ligand; (II) complete structure minimization considering 2000 downstream minimization steps and 6500 steps of conjugate gradient minimization; (III) the minimized systems were progressively heated to 310 K over 0.5 ns, with harmonic restrictions of 10 kcal⋅mol Å^−2^ in the carbon skeleton and ligand; (IV) the system was then balanced for 0.5 ns while adhering to the constraints, and then for 5 ns without constraints to 310 K in a canonical assembly (NVT); and (V) a production dynamic was conducted for 50 ns without constraints at 310 K and 1 atm with a temporary passage of 2 fs using an isothermal isobaric assembly (NPT). In the MD simulation, the temperature was controlled by the Langevin dynamics with a collision frequency of 1 ps^−1^ (NVT) and the pressure with the Berendsen barostat (NPT). In addition, the Particle Mesh Ewald (PME) method with a cut-off value of 10 Å was used to handle nonbonding and long-range electrostatic interactions. All MD simulation calculations were performed using NAMD 2.14, software developed by the Theoretical and Computational Biophysics Group in the Beckman Institute for Advanced Science and Technology at the University of Illinois at Urbana-Champaign [33,34]. Molecular visualization of the systems and MD trajectory analysis were carried out with the VMD 1.9.3 software package [35].

#### Free Energy Calculations

The binding free energy of the hAChE–ligand complexes was estimated using the molecular MM/GBSA technique. For computations, the first 40 ns of MD were extracted and explicit water molecules and ions were removed. Three subsets of each system were analyzed using MM/GBSA: the protein alone, the ligand alone, and the complex (protein–ligand). The total free energy (ΔG_tot_) for each subset was calculated as follows:

$$∆G=E_{MM}+G_{Solv}-{T∆S}_{conf}$$

where E_MM_ represents the bonded and Lennard–Jones energy components; G_Solv_ denotes the polar and nonpolar contributions to the solvation energy; T represents the temperature; and ΔS_conf_ denotes the conformational entropy [36]. Both E_MM_ and G_Solv_ were calculated using the NAMD program with the generalized Born implicit solvent model [37]. ΔG_tot_ was calculated as a linear function of the solvent-accessible surface area, which was calculated with a probe radius of 1.4 Å [38]. The difference between the binding free energies of TcACh and ligand complexes (ΔG_bind_) was used to compute the binding free energy of TcACh and ligand complexes (ΔG_bind_), where values represent the simulation’s averages.

$$∆G_{bind}=G_{tot}\left(complex\right)-G_{tot}\left(protein\right)-G_{tot}(ligand)$$

## 3. Results

### 3.1. Alkaloid Profile

**Figure 3:**
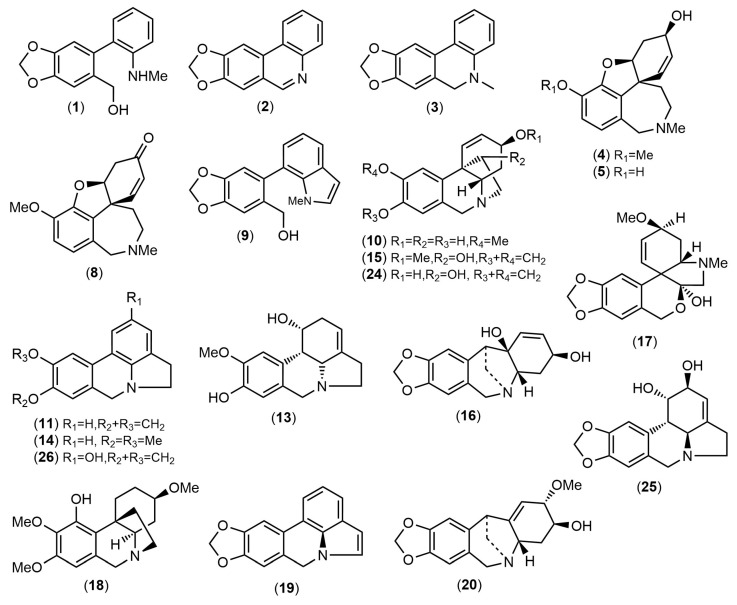
Structures of the alkaloids identified in Amaryllidaceae species collected in Ecuador by GC-MS.

The alkaloid extracts of five samples of Amaryllidaceae collected in Ecuador were analyzed, leading to the quantification of twenty-six alkaloids (Table 1, Figure 3) and six unidentified structures. The amount of each compound are reported in milligrams of galanthamine (mg equivalent GAL) per gram of alkaloid extract (g AE). The chromatograms of these samples are available in the Supplementary Material (Figures S1–S5).

Notable similarities were observed in the alkaloid profile of the two samples of *Urceolina formosa* (collected in Tungurahua’s and Sucumbíos’ provinces) and *Urceolina ruthiana* (Table 1). All these samples presented alkaloids from the galanthamine-, haemanthamine-, and lycorine-type skeletons in their chemical composition. The alkaloid haemanthamine (**15**) was reported in all the *Urceolina* samples evaluated, especially in *U. formosa* (sample B) and *U. ruthiana* (sample C), with 172.5 and 101.4 mg equivalent GAL·g^−1^ AE, respectively. The alkaloids galanthamine (**4**) and 11-hydroxyvittatine (**24**) were found in both *U. formosa* samples (samples A and B), but not in *U. ruthiana*, while lycorine (**25**) was detected in all the *Urceolina* extracts evaluated, with the highest concentrations observed in *U. formosa* (from Sucumbíos province, sample B), followed by *U. formosa* (from Tungurahua province, sample A), with 273.9 and 251.6 mg equivalent GAL·g^−1^ AE, respectively. The alkaloid tazettine (**17**), which belongs to pretazettine-type scaffolds, was reported only in the extract of *U. ruthiana*, with 32.6 mg equivalent GAL·g^−1^ AE. This compound is also known as 6-deoxytazzetine, an artifact derived from the alkaloid pretazettine [40]. No unidentified compound was observed in the species of *Urceolina* evaluated in this study (Table 1).

The alkaloid profile of *Urceolina bonplandii* (previously published as *Eucharis bonplandii*) collected in Colombia showed the presence of structures from narciclasine-, galanthamine-, haemanthamine-, and lycorine-type scaffolds, comprising twelve Amaryllidaceae alkaloids, along with one unidentified compound [41,42]. The same authors also reported the alkaloid composition of *Urceolina caucana* (published as *Eucharis caucana*) collected in two different locations from Colombia—Risaralda and Chocó—describing the presence of narciclasine-, galanthamine-, and lycorine-type alkaloids in both samples, as well as haemanthamine/crinine-, pretazettine-, and montanine-type alkaloids which were observed just in the second species [41].

As shown in Table 1, *C. incarnatus* presented five alkaloids (sanguinine, lycorine, anhydrolycorine, 11,12-dehydroanhydrolycorine, and 2-hydroanhydrolycorine) in its chemical profile, with lycorine (**25**) being the most abundant (149.9 mg equivalent GAL·g^−1^ AE). A significant amount of the compound **26**, currently identified as 2-hydroxyanhydrolycorine by Soto-Vásquez et al. [39], was quantified in this specie, with 60.2 mg equivalent GAL·g^−1^ AE (Table 1).

According to the literature, *C. incarnatus* collected in Peru presented a high amount of lycorine along with seven other different alkaloids [43]. Furthermore, a recent publication revealed the presence of two lycorine-type alkaloids in *C. incarnatus* collected in Ecuador, specifically lycorine and 11,12-dehydroanhydrolycorine, along with four unidentified structures that probably belong to lycorine- and ismine-type skeleton [44].

Consistent with the findings in Table 1, *Stenomesson aurantiacum* (sample E) exhibited the second highest total alkaloids content, at 416 mg equivalent GAL·g^−1^ AE. This plant extract displayed the most diverse chemical profile among the evaluated samples, containing a total of 21 compounds, of which 6 remain unidentified. Based on the fragmentation patterns of the unidentified structures, compound **6** appears to belong to the galanthamine-type scaffold, while compounds **22** and **23** may belong to the pretazettine-type group. The most abundant molecules in this species were 11-hydroxyvittatine (**24**) and galanthamine (**4**), with 108.1 and 74.1 mg equivalent GAL·g^−1^ AE, respectively. Lycorine (**25**) was quantified in all the samples evaluated in this study, except in *S. aurantiacum* (Table 1). In a previous work, 22 alkaloids were detected in *S. aurantiacum* collected in Ecuador, with haemanthamine being the most abundant [15].

### 3.2. AChE and BuChE Inhibitory Activity

**Figure 4:**
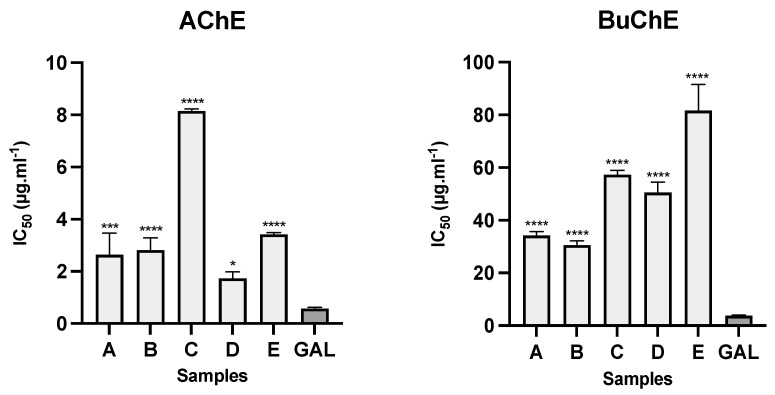
AChE and BuChE inhibitory activity of different species of Amaryllidaceae collected in Ecuador. A: *Urceolina formosa* (from Tungurahua province); B: *Urceolina formosa* (from Sucumbíos province); C: *Urceolina ruthiana*; D: *Clinanthus incarnatus*; E: *Stenomesson aurantiacum*; GAL: galanthamine (control); **** *p* < 0.0001, *** *p* < 0.001, and * *p* < 0.1.

The alkaloid extracts from the bulbs of five samples of Amaryllidaceae collected in Ecuador presented inhibitory activity against AChE and BuChE (Figure 4). The samples from *C. incarnatus* (sample D) and both samples of *U. formosa* (sample A and B) showed the best results toward AChE, with IC_50_ values of 1.73 ± 0.25, 2.63 ± 0.83, and 2.81 ± 0.48 µg·mL^−1^, respectively. For BuChE inhibition, *U. formosa* samples (A and B) had the best results with IC_50_ values of 34.22 ± 1.46 and 30.56 ± 1.56 µg·mL^−1^, respectively, while *C. incarnatus* (sample D) showed IC_50_ values of 50.51 ± 3.93 µg·mL^−1^ against this enzyme. The IC_50_ values for the alkaloid extracts of *U. ruthiana* and *S. aurantiacum* against AChE were 8.15 ± 0.07 and 3.41 ± 0.05 µg·mL^−1^, respectively, and against BuChE, they were 57.26 ± 1.69 and 81.60 ± 9.97 µg·mL^−1^, respectively. Galanthamine, used as a control, showed IC_50_ values of 0.57 ± 0.05 and 3.77 ± 0.20 µg·mL^−1^ against AChE and BuChE, respectively (Figure 4). As shown in Table 1, this alkaloid was observed in the bulb extracts of both samples of *U. formosa* (A and B), and *S. aurantiacum* (E), likely contributing to the cholinesterase inhibitory effect of these species.

The alkaloid profile of *C. incarnatus* revealed the presence of sanguinine at a very low concentration, 3 mg equivalent GAL·g^−1^ AE. According to literature, sanguinine exhibits high activity against AChE [25]. In addition to sanguinine, the alkaloid profile of *C. incarnatus* also presented the compounds anhydrolycorine, 11,12-dehydrolycorine, lycorine, and 2-hydroxyanhydrolycorine. Notably, the last structure was detected in a very high concentration, 60.2 mg equivalent GAL·g^−1^ AE (see Table 1).

According to the literature, the bulb extracts of *U. bonplandii*, *U. caucana* (Risaralda), and *U. caucana* (Chocó) collected in Colombia showed IC_50_ values lower than 10 µg·mL^−1^ against the enzymes *Electrophorus electricus* and *human* AChE and higher than 15 µg·mL^−1^ against horse serum and human BuChE [42]. Among these samples, the *U. bonplandii* bulb extract showed the best results, with IC_50_ values of 0.72 ± 0.05 µg·mL^−1^ against hAChE [42]. Both leaf and bulb extracts of *U. bouchei* Woodson and P.H. Allen collected in Panama showed activity against AChE using the TLC bioautographic method, while the whole plant extract was inactive [45]. A recent publication reported IC_50_ values of the alkaloid extract of *C. incarnatus* above 40 µg·mL^−1^ against AChE and BuChE, indicating low activity against both enzymes [44]. Although the alkaloid profiling of *S. aurantiacum* has been previously documented, this is the first report on its anti-cholinestarase potential.

Previous studies on six species of *Phaedranassa* (Amaryllidaceae) from Ecuador have shown their potential against Alzheimer’s disease [16,17]. According to the literature, *P. cuencana* was the most active against AChE, with IC_50_ values of 0.88 ± 0.11 µg·mL^−1^, while *P. dubia* had the best activity against BuChE, with IC_50_ values of 14.26 ± 2.71 µg·mL^−1^ [16,17]. Recently, alkaloid extracts from the bulbs and leaves of *Crinum* × *amabile* also collected in Ecuador were evaluated against the same enzymes [18]. The bulbs extracts showed the most activity against AChE, with IC_50_ values of 1.35 ± 0.13 µg·mL^−1^, while the leaf extracts had better activity against BuChE, with IC_50_ values of 8.50 ± 0.76 and 45.42 ± 3.72 µg·mL^−1^, respectively [18].

### 3.3. Molecular Docking Results

According to Table 1 and Figure 3, *C. incarnatus* had a high concentration of 2-hydroxyanhydrolycorine (compound **26**), and the best activity against AChE. To understand how compound **26** could work in the inhibition of this enzyme, a theoretical inhibition experiment was conducted through molecular docking, using galanthamine as a control.

We have used the hAChE protein in silico analyses as a theoretical model for biochemical studies of cholinergic neurotransmission [46], anticipating that the next research step could be isolation and inhibition experiments of pure 2-hydroxyanhydrolycorine molecules. Molecular docking results provided binding energies of −8.94 kcal·mol^−1^ for 2-hydroxyanhydrolycorine in comparison to −9.43 kcal·mol^−1^ for galanthamine. As a result, we suggest that 2-hydroxyanhydrolycorine could be as active as galanthamine for two reasons: firstly, the difference between them is only 0.49 kcal·mol^−1^, and secondly, both molecules are localized at the same active site of hAChE. Although the molecular docking experiment can provide an approximate inhibition, a molecular dynamics simulation should be necessary to determine the true behavior within the hAChE active site.

**Figure 5:**
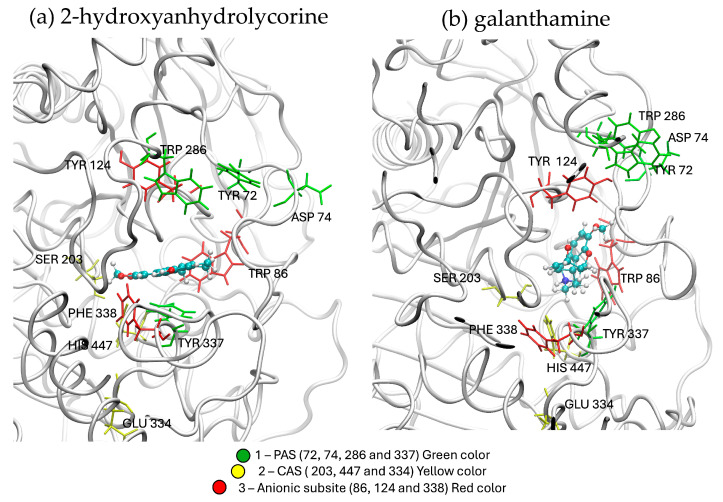
Ligand conformations in the hAChE enzyme obtained by molecular docking experiments.

The hAChE protein’s active site is approximately 20 nm deep and comprises three important regions: (i) the peripheral anionic site (PAS), predominantly composed by Tyr72, Asp74, Trp286, and Tyr337 residues, of the active site gate; (ii) the alpha-anionic site, consisting of Trp86, Phe338, and Tyr124 residues in the middle of the active site; and (iii) the catalytic anionic site (CAS), mainly consisting of Ser203, His447, and Glu 334 residues [47]. The ligand’s position in hAChE enzyme is reported in Figure 5. The 2-hydroxyanhydrolycorine molecule is located at the entrance of the PAS, showing stabilizing interactions with Leu76, Trp86, Phe295, Tyr337, Tyr341, and His447, while galanthamine is located at the catalytic site bottom and shows stabilizing interactions with Trp86, Glu202, Ser203, Phe295, Tyr341, and Tyr337. There are some residues which stabilize both ligands: Trp86, Phe338, and Tyr337, residues belonging to the anionic subsite and CAS. This means both ligands are in the active site of hAChE protein and both could inhibit hAChE. Additionally, experimental reports suggest that Amaryllidaceae alkaloids may exhibit synergistic effects on hAChE inhibition [48].

### 3.4. Molecular Dynamics (MD) Simulations

**Figure 6:**
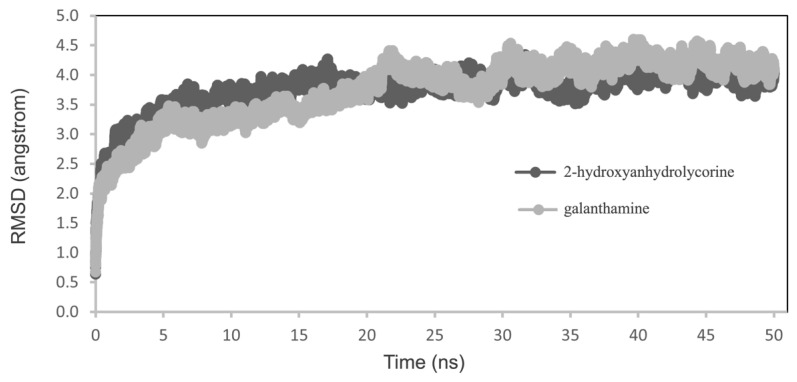
RMSD analysis for 2-hydroxyanhydrolycorine–hAChE and galanthamine–hAChE complexes.

Molecular dynamics simulations were conducted on the poses obtained from molecular docking experiments to understand how 2-hydroxyanhydrolycorine inhibits hAChE. The free energy of binding for both complexes was calculated using molecular mechanics generalized Born solvent accessibility (MM-GBSA). A small variation in root-mean-square deviation (RMSD) indicates the stability of the protein–ligand conformations during the MD simulations (Figure 6).

The free energies of binding calculated by MM-GBSA, Table 2, indicate that the 2-hydroxyanhydrolycorine complex is significantly less stable than the galantamine complex (approximately 13 kcal·mol^−1^). Although these results indicate a difference in inhibitory potency between them, they do not indicate that 2-hydroxyanhydrolycorine molecule does not inhibit. MMGBSA is a computational method and does not perfectly capture all the complexities of real-world binding. Factors beyond binding energy, such as inhibitor flexibility or pocket dynamics, or assumptions about solvation (effects of water molecules) can affect accuracy. Overall, further studies such as experimental inhibition assays are needed to confirm the true difference in potency.

**Figure 7:**
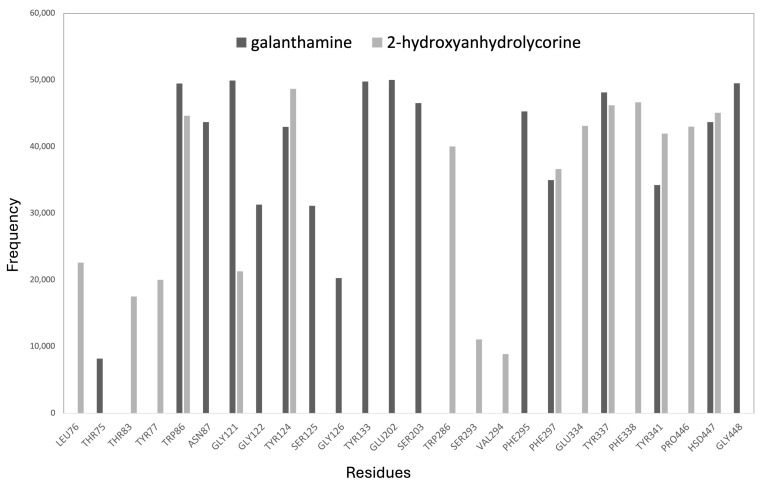
Residue occurrence frequencies at 3 Å or less from the ligand within the ligand–hAChE complex calculated by molecular dynamics procedures.

A frequency graph of contact between the most significant residues and the 2-hydroxyanhydrolycorine and galanthamine molecules during the MD is presented in Figure 7. A radius of 3 Å was chosen as this is the average distance for hydrogen bond variations in the analyzed systems. The MD results show that galanthamine remains mostly at the bottom of the active site, near the Asn87, Ser125, Gly126, Tyr133, Glu202, Ser203, and Phe295 residues. In contrast, 2-hydroxyanhydrolycorine is localized at the PAS site near the Leu76, Thr83, Tyr77, Trp286, Ser293, Val294, Glu334, Phe338, and Pro446 residues. Figure 7 shows that both ligands have a significant frequency of contact with the Phe297 residue, which is involved in the formation of the acyl-binding pocket.

## 4. Conclusions

In summary, this study presents the first report on the alkaloid profiling of *Urceolina* species collected in Ecuador, as well as the first investigation into the anti-cholinesterase potential of the species studied, except for *C. incarnatus*. The extract of *Stenomesson aurantiacum* revealed unidentified structures, suggesting that this species is a potential source of new Amaryllidaceae alkaloids. Among the samples studied, *C. incarnatus* and *Urceolina formosa* (collected in Sucumbíos province) provided the best results against AChE and BuChE, respectively. Computational experiments suggest that the interactions between 2-hydroxyanhydrolycorine and hAChE are similar to those observed for galanthamine. Further in vitro assays with 2-hydroxyanhydrolycorine are necessary to corroborate the in silico findings. Our results highlight the importance of Amaryllidaceae species in the search for new bioactive molecules.

## Figures and Tables

**Table 1:** Alkaloids identified in Amaryllidaceae species collected in Ecuador by GC-MS. Values are expressed as mg equivalent GAL·g^−1^ AE.

Alkaloid	RT	RI	MS	A	B	C	D	E
ismine (**1**)	21.34	2304.2	257(42), 239(20), 238(100), 196(8), 168(7)	-	-	-	-	2.8
trisphaeridine (**2**)	21.65	2323.1	223(100), 222(39), 164(11), 138(16), 111(6)	-	-	-	-	8.1
5,6-dihydrobicolorine (**3**)	22.35	2362.2	239(57), 238(100), 180(9), 90(4), 89(1)	-	-	-	-	4.3
galanthamine (**4**)	23.43	2386.1	287(92), 286 (100), 270(15), 244(28), 216(34)	46.2	28.2	-	-	74.1
sanguinine (**5**)	23.71	2452.4	273(100), 272(72), 256(17), 202(21), 160(25)	-	-	-	3.0	7.8
unidentified (galanthamine-type) ^1^ (**6**)	24.06	2474.4	287(55), 286(100), 256(45), 228(29), 211(9)	-	-	-	-	24.0
unidentified (**7**)	24.27	2481.4	301(65), 300(100), 284(14), 270(41), 242(23)	-	-	-	-	5.1
narwedine (**8**)	24.60	2509.7	285(91), 284(100), 242(14), 216(21), 174(23)	-	-	-	-	3.4
galanthindole (**9**)	24.99	2535.0	281(100), 262(18), 252 (13), 225(5) 191(8)	-	-	-	-	15.8
8-*O*-demethylmaritidine (**10**)	25.07	2539.9	273(100), 254(6) 230(19), 201(67), 189(35)	-	-	5.5	-	6.1
anhydrolycorine (**11**)	25.12	2543.6	251(49), 250(100), 192(10), 191(9), 163(2)	-	-	-	24.5	-
unidentified (**12**)	25.62	2576.1	301(100), 286(30), 270(24), 203(32), 174(50)	-	-	-	-	45.4
kirkine (**13**)	25.83	2590.1	253(64), 252(100), 237(19), 209(14), 280(4)	-	-	-	-	23.1
assoanine (**14**)	26.10	2608.1	267(61), 266(100), 250(23), 126(3)	-	-	-	-	3.3
haemanthamine (**15**)	26.14	2611.5	301(11), 272(100), 240(16), 199(9), 181(28)	172.5	37.2	101.4	-	-
pancratinine C (**16**)	26.32	2613.5	287(99), 203(51), 188(64), 176(100), 174(85)	-	-	-	-	4.9
tazettine (**17**)	26.51	2620.4	331(23), 316(12), 298(19), 247(100), 201(20)	-	-	32.6	-	-
hippeastidine (**18**)	26.64	2625.8	319(100), 304(19), 288(33), 246(14), 233(46)	-	-	-	-	41.7
11,12-dehydroanhydrolycorine (**19**)	26.65	2644.4	249(68), 248(100), 191(8), 190(19), 1985)	-	-	-	4.9	18.4
montanine (**20**)	26.85	2646.5	301(100), 271(13), 270(63), 257(31), 226(20)	-	-	-	-	3.6
unidentified (**21**)	27.00	2650.1	349(65), 334(50), 318(100), 291(52), 232(42)	-	-	-	-	4.0
unidentified (pretazettine-type) ^1^ (**22**)	27.39	2697.3	331(52), 300(15), 261(100),228(24), 201(16)	-	-	-	-	8.2
unidentified (pretazettine-type) ^1^ (**23**)	27.65	2795.6	331(25), 261(100), 228(23), 197(8), 169(6)	-	-	-	-	3.8
11-hydroxyvittatine (**24**)	28.31	2716.5	287(7), 259(19), 258(100), 242(9), 211(13)	16.9	9.8	-	-	108.1
lycorine (**25**)	29.10	2779.4	287(22), 268(19), 250(12), 227(62), 226(100)	251.6	273.9	168.1	149.9	-
2-hydroxyanhydrolycorine ^2^ (**26**)	30.20	2891.6	267(56), 266(100), 236(4), 208(9), 132(6)	-	-	-	60.2	-
**Total**				**487.2**	**349.1**	**307.6**	**242.5**	**416.0**

^1^ proposed alkaloid-type according to fragmentation pattern; ^2^ proposed alkaloid structure according to fragmentation pattern by [39]; RT: retention time; RI: retention index; MS: mass spectra; A: *Urceolina formosa* (from Tungurahua province); B: *Urceolina formosa* (from Sucumbíos province); C: *Urceolina ruthiana*; D: *Clinanthus incarnatus*; E: *Stenomesson aurantiacum*; MS: mass spectra; RI: retention index.

**Table 2:** Free energy calculations of ligand–hAChE complexes calculated through molecular dynamics and molecular docking procedures.

Substrate	MM-GBSA (kcal·mol^−1^)	Molecular Docking (kcal·mol^−1^)
2-hydroxyanhydrolycorine	−23.695 (2.106)	−8.94
galanthamine	−36.710 (2.842)	−9.43

## Data Availability

Data are contained within the article.

## Supplementary Materials

The following supporting information can be downloaded at: https://www.mdpi.com/article/10.3390/life14080924/s1, Figure S1: GC chromatogram of the alkaloid extract of *Urceolina formosa* (from Tungurahua province), sample A; Figure S2: GC chromatogram of the alkaloid extract of *Urceolina formosa* (from Sucumbíos province), sample B; Figure S3: GC chromatogram of the alkaloid extract of *Urceolina ruthiana*, sample C; Figure S4: GC chromatogram of the alkaloid extract of *Clinanthus incarnatus*, sample D; Figure S5: GC chromatogram of the alkaloid extract of *Stenomesson aurantiacum*, sample E.
