# Supplementary material for: Alkaloid Profiling and Anti-Cholinesterase Potential of Three Different Genera of Amaryllidaceae Collected in Ecuador: Urceolina Rchb., Clinanthus Herb. and Stenomesson Herb
Source: Life (Basel). 2024 Jul 24;14(8):924. doi: 10.3390/life14080924 (PMC11355869; doi:10.3390/life14080924)
Supplement: Supplementary file 1 [file life-14-00924-s001.zip › life-3105510-Supplementary Material.pdf]

Supplementary Material

Alkaloid profiling and anti-cholinesterase potential of three different genera of Amaryllidaceae collected in Ecuador: *Urceo-lina* Rchb., *Clinanthus* Herb., and *Stenomesson* Herb.

Luciana R. Tallini [1]; Karen Acosta León [2]; Raúl Chamorro [2]; Edison H. Osorio [3]; Jaume Bastida [1]; Lou Jost [4], Nora H. Oleas [5,*]

[1] Grup de Productes Naturals, Departament de Biologia, Sanitat i Medi Ambient, Facultat de Farmàcia i Ciències de l'Alimentació, Universitat de Barcelona, Av. Joan XXIII 27-31, 08028 Barcelona, Spain; ruscheltallini@ub.edu
[2] Grupo de Investigación de Productos Naturales y Farmacia, Facultad de Ciencias, Escuela Superior Politécnica del Chimborazo, Panamericana Sur km 1 1/2, EC060155 Riobamba, Ecuador;
[3] Facultad de Ciencias Naturales y Matemáticas, Universidad de Ibagué, Carrera 22 calle 67, Ibagué, Colombia;
[4] Fundación Ecominga, Vía a Runtún s/n, Baños, Tungurahua, Ecuador;
[5] Centro de Investigación de la Biodiversidad y Cambio Climático (BioCamb) y Facultad de la Salud y Bienestar Humano, Universidad Tecnológica Indoamérica, Machala y Sabanilla, EC170301 Quito, Ecuador.
* Correspondence: noraoleas@uti.edu.ec

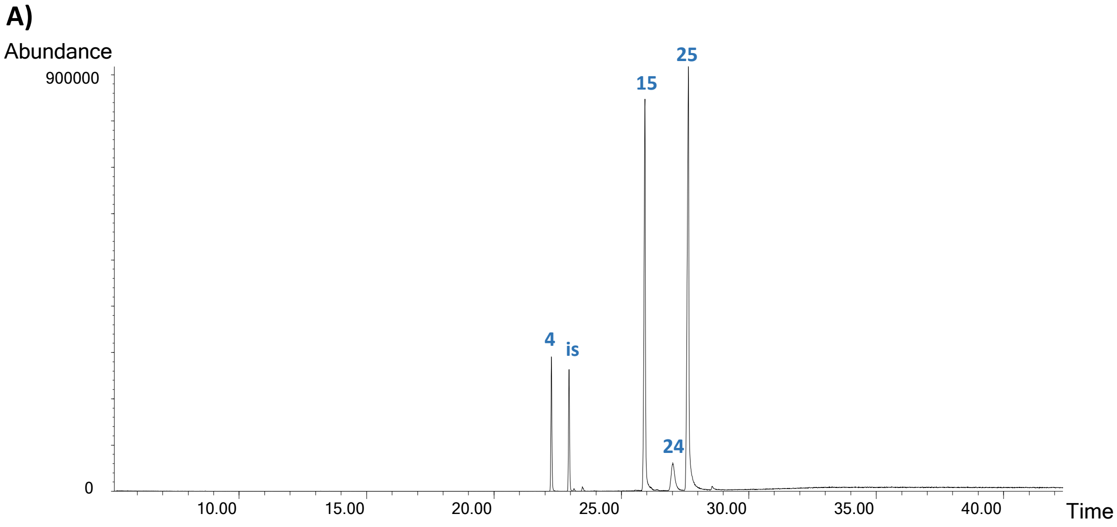

Figure S1. GC chromatogram of the alkaloid extract of *Urceolina formosa* (from Tungurahua province), sample A.

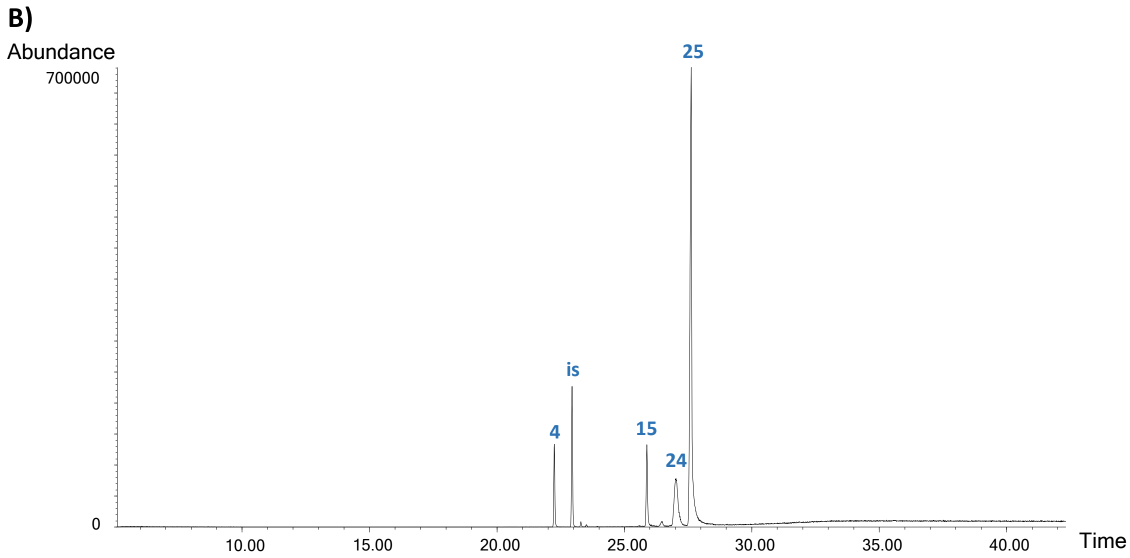

Figure S2. GC chromatogram of the alkaloid extract of *Urceolina formosa* (from Sucumbíos province), sample B.

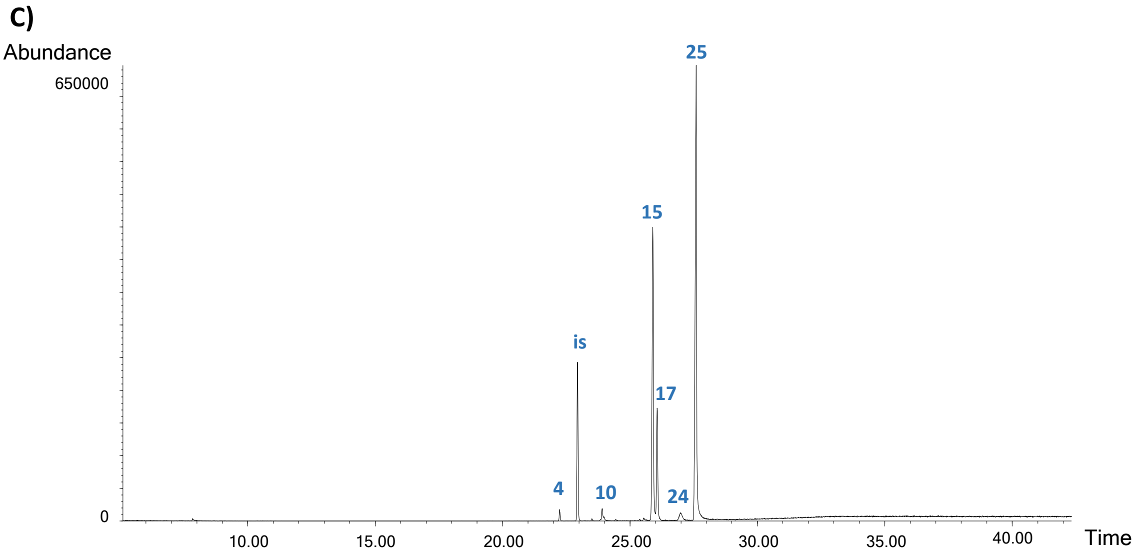

Figure S3. GC chromatogram of the alkaloid extract of *Urceolina ruthiana*, sample C.

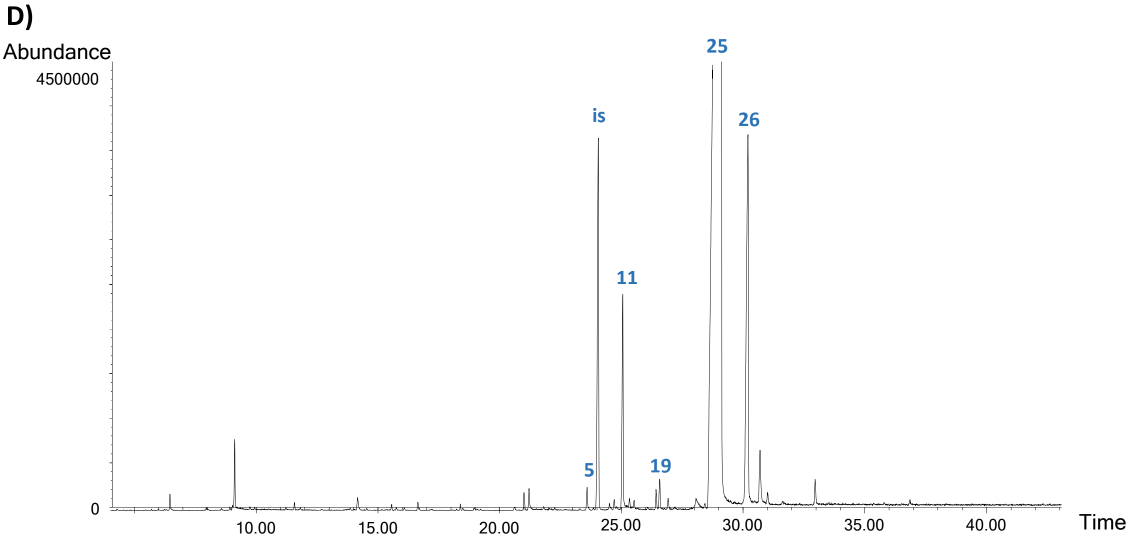

Figure S4. GC chromatogram of the alkaloid extract of *Clinanthus incarnatus*, sample D.

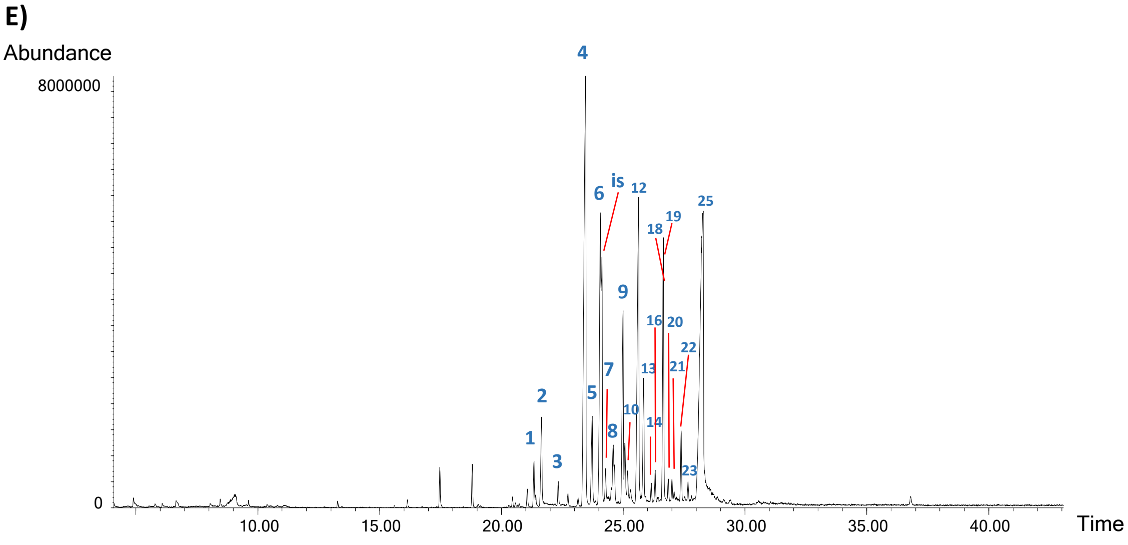

Figure S5. GC chromatogram of the alkaloid extract of *Stenomesson aurantiacum*, sample E.
